# dingo: a Python package for metabolic flux sampling

**DOI:** 10.1093/bioadv/vbae037

**Published:** 2024-03-22

**Authors:** Apostolos Chalkis, Vissarion Fisikopoulos, Elias Tsigaridas, Haris Zafeiropoulos

**Affiliations:** GeomScale.org; GeomScale.org; Department of Informatics & Telecommunications, National and Kapodistrian University of Athens, Panepistimioupolis, Ilisia,16122 Athens, Greece; GeomScale.org; Inria Paris and IMJ-PRG, Sorbonne Université and Paris Université, France; GeomScale.org; Laboratory of Molecular Bacteriology, Department of Microbiology, Immunology and Transplantation, Rega Institute for Medical Research, KU Leuven, 3000 Leuven, Belgium

## Abstract

We present dingo, a Python package that supports a variety of methods to sample from the flux space of metabolic models, based on state-of-the-art random walks and rounding methods. For uniform sampling, dingo’s sampling methods provide significant speed-ups and outperform existing software. Indicatively, dingo can sample from the flux space of the largest metabolic model up to now (Recon3D) in less than a day using a personal computer, under several statistical guarantees; this computation is out of reach for other similar software. In addition, dingo supports common analysis methods, such as flux balance analysis and flux variability analysis, and visualization components. dingo contributes to the arsenal of tools in metabolic modelling by enabling flux sampling in high dimensions (in the order of thousands).

**Availability and implementation:**

The dingo Python library is available in GitHub at https://github.com/GeomScale/dingo and the data underlying this article are available in https://doi.org/10.5281/zenodo.10423335.

## 1 Introduction

Metabolic models enhance the study of the relationship between genotype and phenotype in an attempt to elucidate the mechanisms that govern the physiology and the growth of a species and/or a community (Morris *et al.* 2020). By optimizing a linear objective function over a polytope, flux balance analysis (FBA) identifies a single optimal flux distribution (Orth *et al.* 2010). Flux variability analysis (FVA) reveals the limits of the solution space (Gudmundsson and Thiele 2010). Contrary to FBA and FVA, flux sampling is an unbiased method, as it does not depend on the selection of the objective function. It allows us to cover all the possible flux values by estimating a probability distribution for the flux value of a certain reaction (Schellenberger and Palsson 2009).

The ability to sample (efficiently) points from the convex polytope corresponding to (the steady states of) a metabolic model allows us to investigate its whole solution space. This way, we can obtain a more detailed insight of a system at steady state; where the production rate of each metabolite equals its consumption rate. Alternatively, we can perform flux sampling after optimizing an objective function, and approximate the flux distributions in optimal scenarios.

Even though flux sampling has proved itself by delivering great insights in a range of applications (Herrmann *et al.* 2019), high dimensionality- and anisotropy-oriented limitations (Schellenberger and Palsson 2009) force the current implementations to struggle or even to fail in several cases (Fallahi *et al.* 2020). A range of Markov chain Monte Carlo (MCMC) algorithms and implementations have been developed to address this obstacle (Fallahi *et al.* 2020) (see Supplementary File—Section 1). In this setting, we present dingo, a Python package that supports efficient flux sampling, based on a variety of state-of-the-art MCMC sampling algorithms; it also provides classical FBA and FVA methods and advanced visualizations.

## 2 Implementation

dingo is an open-source Python package that exploits the efficiency of volesti, an open-source C++ software library that implements high-dimensional MCMC sampling and volume approximation algorithms.

dingo supports a variety of MCMC algorithms for uniform sampling. Among them, the multiphase Monte Carlo sampling (MMCS) algorithm (Chalkis *et al.* 2021) has been reported as the most efficient algorithm in practice. MMCS unifies rounding and sampling of a convex polytope in one pass, obtaining both upon termination. In this study, we show that combining the rounding of PolyRound with the optimized Billiard walk implementation of dingo, i.e. Billiard walk with rounding (BWR), yields the fastest sampling for the networks we test (up to dimension 5335). Interestingly, we show that as the networks’ dimension increases, MMCS will overrule. An example of an application that creates polytopes in higher dimensions is sampling from the solution space of community models where several metabolic networks are combined.

dingo enables the performance of the MMCS algorithm in parallel threads and uses the state-of-the-art linear programming solvers of GurobiGurobi Optimization, LLC (2023). It ensures the quality of the output samples using two widely used diagnostics, the effective sample size (ESS) (Geyer 1992) and the potential scale reduction factor (PSRF) (Gelman and Rubin 1992); dingo guarantees bounded values for both diagnostics for the returned sample. In addition to the MMCS algorithm and the optimized Billiard walk, it also supports the random directions hit-and-run (Smith 1984), the coordinate directions hit-and-run (CDHR) (Smith 1984), the Dikin (Kannan and Narayanan 2012), the John and Vaidya (Chen *et al.* 2018), and the ball walk (Lovász *et al.* 1997) sampling algorithms.

We ensure the correctness of dingo’s functionality using a set of unit tests running on a continuous integration platform. All three main formats for metabolic models (.xml, .json, and .mat) are supported. A tutorial is available as a Google Colab notebook.

## 3 Performance comparison and illustrations

**Figure 1.**
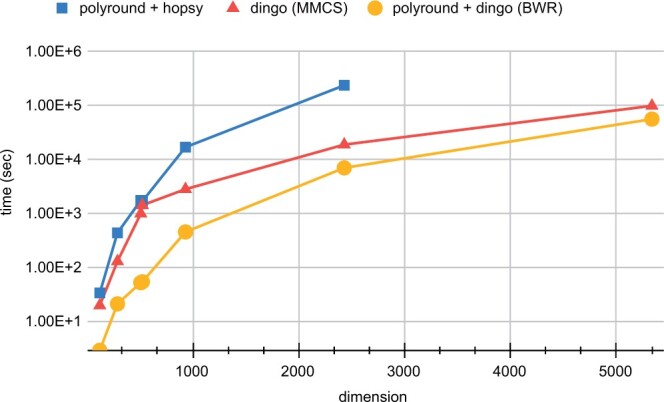
Comparison of three sampling methods (PolyRound with hopsy, dingo’s MMCS and PolyRound with dingo’s BWR) when sampling from the flux space of seven GEMs corresponding to polytopes of dimension ranging from 122 to 5335, under the same statistical guarantees. PolyRound was used for rounding with hopsy and dingo’s Billiard walk used to sample from the rounded polytope. dingo’s MMCS run-time corresponds to both rounding and sampling, starting from the non-rounded polytope (i.e. same as the input of PolyRound).

Currently, the most efficient way to perform flux sampling, to the best of our knowledge, is to combine the PolyRound Python package (Theorell *et al.* 2021) (for rounding the polytope) with CDHR sampling algorithm as implemented in the HOPS C++ library (Jadebeck *et al.* 2021) to sample from the rounded polytope. We compare two sampling methods implemented in dingo against the combination of PolyRound and hopsy (the Python interface of HOPS), over a set of models having dimension from around 100 to more than 13 000. In all cases, we perform a pre-processing step using PolyRound. dingo performs rounding and sampling in one step using the MMCS algorithm or using an optimized Billiard walk to sample from the rounded polytope obtained by PolyRound (see also Supplementary File—Section 2), i.e. BWR. To ensure that the quality of the sample provides an accurate approximation of the target distribution, we require an ESS of 1.000 and a PSRF of at most 1.1, in all cases. To our knowledge, dingo is the only software that provides this combined statistical guarantee. For all the tested models dingo is faster than PolyRound/hopsy. Moreover, dingo’s added value highlights as the model’s dimensions increases (see Fig. 1). Indicatively, dingo can sample the latest version of the human metabolic network, the Recon3D model (Brunk *et al.* 2018), in less than 16 h, using modest hardware; while after 10 days, hopsy did not converge.

To demonstrate dingo’s flux sampling and illustrations tools in a real-world scenario, we use the integrated human alveolar macrophage model with the virus biomass objective function of Sars-Cov-2 (Renz *et al.* 2020) (see Supplementary File—Section 3). Notably, our findings confirm the authors’ indicating Guanylate Kinase 1 as a potential therapeutic target.

## 4 Conclusions

dingo is a Python package that employs efficient C++ MCMC implementations from volesti library. It supports a variety of MCMC algorithms and classical methods as FBA and FVA. dingo unlocks the fastest implementation for sampling current metabolic networks namely BWR. Additionally, dingo provides an implementation of the MMCS algorithm that is also more efficient that the current state-of-the-art but also our experiments denote that it would be faster than BWR for higher dimensions. It also offers statistical and illustration tools, like copula estimation, that can help the user to extract useful information about the model (see Supplementary File—Section 3). dingo facilitates the survey of the largest models available for the time being assuring for the first-time high quality of the samples returned. Moreover, it requires minimum computational resources requirements and aims to support a broad spectrum of research and application needs via a user-friendly design.

## Supplementary Material

vbae037_Supplementary_Data
