# Supplementary material for: dingo: a Python package for metabolic flux sampling
Source: Bioinform Adv. 2024 Mar 22;4(1):vbae037. doi: 10.1093/bioadv/vbae037 (PMC10997433; doi:10.1093/bioadv/vbae037)
Supplement: vbae037_Supplementary_Data [file vbae037_supplementary_data.pdf]

dingo: a Python package for metabolic flux sampling
Supplementary material

Apostolos Chalkis, Vissarion Fisikopoulos, Elias Tsigaridas, and Haris Zafeiropoulos

March 1, 2024

1 Convex polytope sampling algorithms and available implementations

Uniform sampling from the interior of high dimensional polytopes is a very challenging from a computational point-of-view. Nevertheless, it has a wide range of applications, among them the flux space of metabolic networks, and thus it is an active area of research in computational geometry and statistics; several different sampling algorithms have been developed over the years and are consequently implemented in various software packages (Table 1).

The majority of the algorithms and their corresponding implementations exploit Markov Chain Monte Carlo (MCMC) methods, as MCMC sampling is the most efficient choice for high dimensional problems [20]. The crux of MCMC consists of random walks inside the polytope. In this setting, the widely used hit-and-run family of random walks generates a chain of points by taking steps of random length inside the polytope in randomly generated directions. Each variant in this family generates the set of possible directions in a different way, e.g., Coordinate Hit-and-Run, at each step picks randomly a line parallel to the axis and then a random point on the part of the line that lies inside the polytope. Another family of walks are the affine-invariant ones, namely the Dikin [21], Vaidya, and John Walk [8]. These walks select the new point from the interior of an inscribed to the polytope ellipsoid. Each variant in this family defines the corresponding ellipsoid differently. The GAPSPLIT algorithm tries to divide the solution space uniformly to calculate random samples. To support flux sampling on the solution space of metabolic models, several software packages exists that implement these algorithms and also provide interfaces for a variety of languages, such as MATLAB (`cobra` [16, 17], `gapsplit` [22], optGpSampler [26]), Python (`cobra`, `gapsplit`, `optGpSampler`, `HOPS` [18]), C++ (`HOPS`, `polytopewalk` [8] [1]). The most efficient algorithm today for sampling polytopes derived from metabolic networks is reported to be the Coordinate Hit-and-Run with Rounding (CHRR) [18], while it has been the algorithm of choice for the state-of-the-art software (see `HOPS` [18] and `cobra` [17]). CHRR, before sampling, applies a pre-processing step, called rounding, so that the efficiency of the random walk in the sampling phase is improved. For sampling it employs the Coordinate Hit-and-Run (CHR) algorithm.

Typically, rounding is applied on the polytope before sampling to improve its roundness. Roughly speaking, there are two notions of roundness: a polytope in John position [19] and a well-rounded polytope [25]. Both contain the unit ball, while the first is contained in a ball of radius $\widetilde{O}(n)$ and the second in a ball of radius $\widetilde{O}(\sqrt{n})$ where the $\widetilde{O}(\cdot)$ notation implies that we ommit the logarithic factors. We know that a polytope in isotropic position [28] is also well-rounded. Despite the fact that John position, in the worst case, is worse than well roundness, it is the main choice in the existing software for rounding because it is computationally easier to achieve. To the best of our knowledge, the Python package `PolyRound` provides the most efficient implementation to achieve John position by computing the largest inscribed ellipsoid in the polytope and apply to it the transformation that maps the ellipsoid to the unit ball (`cobra` and `HOPS` also implement this rounding method).

[1] `https://github.com/yuachen/polytopewalk`

Sampling algorithm	Software					
	`cobra` [16, 17]	`HOPS` [18]	polytopewalk [8]	gapsplit [22]	optGpSampler [26]	`dingo`
GAPSPLIT [22]				x		
Random Directions Hit-and-Run [35]	x	x				x
Artificial Centering Hit-and-Run [27, 17]	x				x	
Coordinate Hit-and-Run []	x	x				x
Dikin Walk [21]		x	x			x
Vaidya [8]			x			x
John Walk [8]			x			x
Ball Walk [24]			x			x
Billiard Walk [15]						x
CHRR* [16]	x	x				x
Multiphase Monte Carlo Sampling* [6]						x

Table 1: Selection of convex polytope sampling algorithms and related software packages. We denote with asterisk (*) the algorithms that also perform rounding to the polytope.

Our contribution, a Python library called `dingo`, supports a suite of sampling approaches (Table 1) initially implemented in C++ as parts of the `volesti` library [5]. The optimized implementation of Billiard Walk [15] (BW) supported in `dingo` employs boundary reflections to outperform the mixing time of Hit-and-Run algorithms in practice [4, 6]. This implementation of BW achieves a reduced cost per step compared to previous BW implementations [15, 9, 37] and it is faster by a factor of n for convex polytopes.

Additionally, `dingo` provides Multiphase Monte Carlo Sampling (MMCS) [6], and isotropic rounding that, to our knowledge, are not available at any other software package. In brief, MMCS constructs a sequence of polytopes (phases) such that sampling is accelerated in each phase. In each phase, MMCS samples from the corresponding polytope and then applies to it a linear transformation that maps the sample to an isotropic position, to obtain the polytope of the next phase. All the samples are mapped back to the initial polytope. Thus, MMCS obtains both a uniform sample and a polytope in near isotropic position upon termination. MMCS is the first method that unifies rounding and sampling in one pass and it is typically applied on a non-rounded polytope. Therefore, the rounding preprocess is not necessary for MMCS. A parallel implementation of MMCS is also supported.

To guarantee the quality of the samples `dingo` computes the total Effective Sample Size (ESS) and the overall Potential Scale Reduction Factor (PSRF) during sampling and stops when both achieve a certain threshold given by the user.

2 Performance comparisons

To illustrate the efficiency of `dingo` we compare its implementations against the best existing implementation of CHRR[2]. `HOPS` library is reported to be six times faster for sampling than `cobra` [18] while both implementing CHRR. However, since `PolyRound` provides the most efficient implementation for the rounding step in CHRR, we compare against the combination of `PolyRound` and `HOPS`. In particular, we compute the rounded polytope with `PolyRound` and we sample from the rounded polytope using the CHR impementation in `hopsy`, the python API of `HOPS` (Figure 1 top box and Figure 2 red line)[3] .

We test CHRR as described above against the execution times with `dingo`'s implementations. First, we sample from the rounded polytopes obtain by `PolyRound` using the `dingo`'s BW, we call it BWR (Figure 1 intermediate box and Figure 2 green line). Finally, we sample from the non-rounded polytope using the MMCS implementation of `dingo` (Figure 1 bottom box and Figure 2 blue line). To obtain the non-rounded polytope corresponding to a metabolic network, we use the routines of `PolyRound` as it also provides efficient facet redundancy removal implementations (Figure 2 yellow line). This step was common in all three sampling methods we compared.

[2] The evaluation of the efficiency of the CHRR implementation in `dingo` is out of the scope of this paper.
[3] `https://github.com/modsim/hopsy`

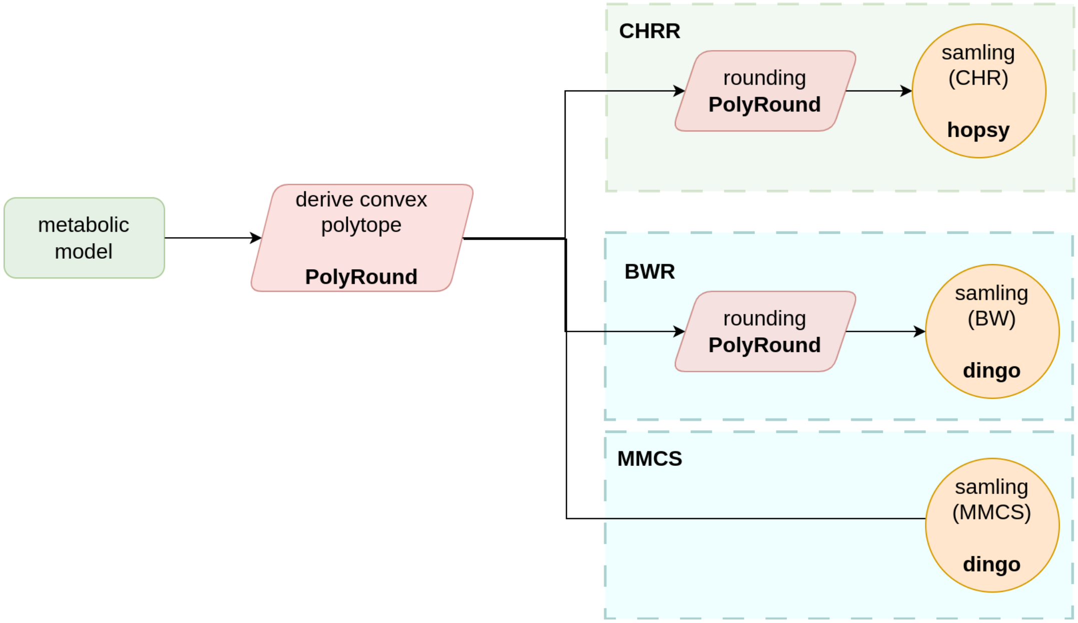

Figure 1: Workflow followed for comparing `dingo` with state-of-the-art sampling approaches. In blue boxes the approaches using the `dingo` Python library. CHR: Coordinate Hit-and-Run; CHRR: CHR Rounded; BW: Billiard Walk; BWR: BW Rounded; MMCS: Multiphase Monte Carlo Sampling.

		PolyRound	hopsy		dingo		dingo
**Model name**	**d**	**rounding**	**CHR**	**CHRR**	**BW**	**BWR**	**MMCS**
iJN746	122	2	31	33	1	3	19
iAT_PLT_636	289	15	421	437	6	21	129
iSDY_1059	509	37	1707	1744	15	52	993
iAF1260	524	37	1651	1688	17	54	1422
recon1	931	357	16535	16892	97	454	2817
recon2d	2430	5817	230636	236453	1174	6991	18855
recon3d	5335	50119	NA	NA	5842	55961	98620

Table 2: Time (in seconds) performance for sampling a metabolic model with `hopsy` and `dingo` packages. Rounding for CHRR and BWR is performed by `PolyRound`. The dimension d is after preprocessing. For `hopsy` we use a thinning of $100d$ for all models but Recon3D where we use $200d$ as suggested by the authors [18]. NA means that after 10 days, `hopsy` was not able to converge and the process stopped at ESS=430, PSRF=1.024.

We perform our benchmarks by requesting ESS $= 1000$ and PSRF ≤ 1.1 for `dingo`. We pick these values for illustration reasons as we analyze the progress of both ESS and PSRF during the run of the sampling methods later (Figure 3). Since `hopsy` does not have an option to request a certain value of ESS we count its run-time until the generated sample achieves the same targets for both ESS and PSRF as in `dingo`. Last, in `hopsy` we use 5 chains following the same process as in [18]. Our benchmark dataset constitutes of five models from the BiGG Models knowledgebase that correspond to polytopes of dimension ranging from 122 to 5335 including Recon2 (version 2.2) from the BioModels database and Recon3D from Virtual Metabolic Human database. The results are illustrated in Table 2 and in Figure 2.

Notably, both `dingo`'s implementations of BWR and MMCS are faster than CHRR. BWR is is 11-33 times faster than CHRR while MMCS is 2-13 times faster depending on the dimension. BWR is faster than MMCS for the benchmarks in the Table 2. However, the gap between the

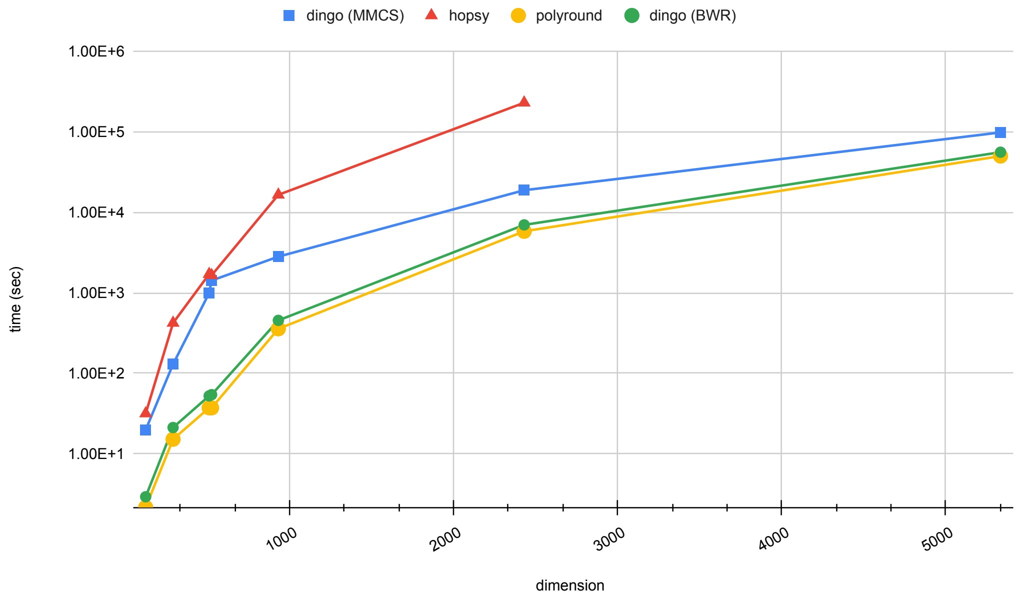

Figure 2: Runtime comparison of the three sampling methods in Table 2. The runtime of `PolyRound` corresponds to the preprocess step of obtaining and rounding the polytope that corresponds to each metabolic model.

MMCS and BWR runtimes decreases as the dimension of the polytope increases denoting that MMCS will eventually outperform BWR for higher dimensional polytopes that correspond to bigger models. This result can be explained by the analysis of algorithms that compute the largest inscribed ellipsoid of a polytope [23, 36] where the performed operations increase faster as the dimension increases than the performed operations in MMCS [6]. Moreover, MMCS achieves a better rounding than `PolyRound` in its last phases, and thus, BW mixing rate increases. The need for enabling flux sampling in even higher dimensions in the analysis of metabolic models is certain especially as community models emerge; a community model consists of a set of metabolic networks and there are several approaches to build such a model [14]. Last but not least, `dingo` can sample from recon3D and obtain an ESS = 1000 in less than 16 hours, while `hopsy` could not reach that ESS in 10 days. For details see Table 2.

For the reproducibility of our benchmark results note that we have used `dingo` version 0.1.0[4], `PolyRound` version 0.2.0 and `hopsy` version v0.2.0[5]. Also the scripts that we have used along with their outcomes (processed polytopes and samples) are publicly available under the following Zenodo repository [7]:

https://zenodo.org/records/10423335.

Figure 3 illustrates the progress of ESS and PSRF in all the three sampling methods we compare for the model iSDY_1059. To ensure a meaningful comparison, we report ESS and PSRF values at the points where the MMCS transitions to the next phase based on the proportion of points sampled. Specifically, MMCS undergoes four phases, transitioning at 29%, 58%, and 87% of the total generated points, respectively. The reported ESS and PSRF values for the other two methods are synchronized with MMCS's progression. Notably, both BWR and CHRR exhibit similar fluctuations in ESS and PSRF, yet BWR's superior convergence rate results in faster runtimes. In contrast, MMCS demonstrates less efficiency in the initial two phases compared to the latter two. This inefficiency is attributed to the time spent rounding the polytope during the

[4] commit hash #$03d6e65a1278753bd879ea24f6512e7e8b95af93$

[5] commit hash #$4ad62ade6aa841d37f35d24e68043b3581358ce6$

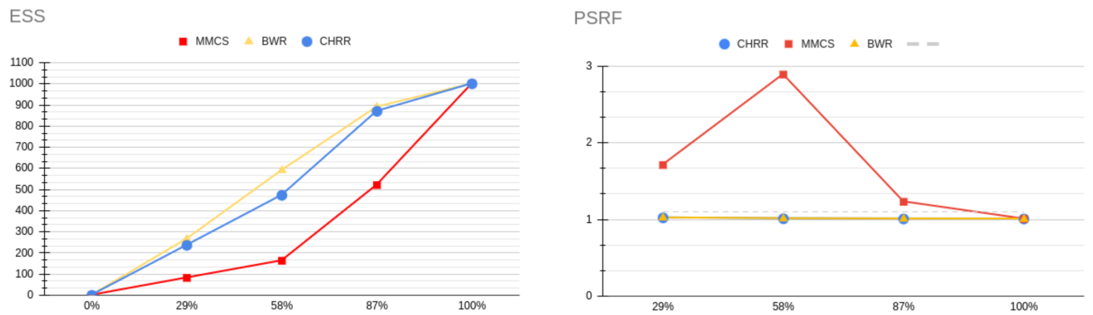

Figure 3: The progress of Effective Sample Size (ESS) and Potential Scale Reduction Factor (PSRF) for the three sampling methods for the model iSDY_1059. The percentages at the x-axis correspond to proportions of the total number of samples when MMCS transits from a phase to the next one. We report the values for all the three models on the same proportion of points to obtain a meaningful comparison.

initial phases. Once sufficiently rounded, MMCS exhibits a rapid increase in performance during the later phases.

3 Illustrations and statistical tools

`dingo` provides some illustrations and statistical tools to help the user to make inference on a metabolic model. In particular, it provides probability density estimation for a flux distribution, given the marginal samples, and copula estimation and plotting. A copula is a bivariate probability distribution for which the marginal probability distribution of each variable is uniform; it can capture the dependency between two random variables (e.g., two reaction fluxes).

Recently, the virus biomass objective function (VBOF) of SARS-CoV-2 was generated and integrated with a genome-scale metabolic model of human alveolar macrophages by Renz *et al.* [31]. In their study, the authors performed Flux Balance Analysis (FBA) combined with reactions knock-out to reveal that the knock-out of Guanlyate Kinase 1 (GK1) decreased the growth of the virus to zero, while not affecting the one of the host. Among its several approaches [34], flux sampling can be used to increase the confidence level of FBA predictions [29]. As a demonstration, we performed flux sampling with `dingo` using the integrated human-virus model.

The flux space of the model was first sampled in an unbiased way, i.e., without requiring any optimization function to be optimized. Sampling was also performed after maximizing the host's biomass function and finally, after maximizing the VBOF. In each of these cases, a flux value distribution for each reaction of the model was obtained, instead of a single value as in the FBA case. The plots in Figure 4 illustrate the distributions of the flux values of the Tyramine Sulfotransferase (TYMSULT) and the Guanylate Kinase 1 (GK1) reactions after maximizing for the host's biomass function (Figure 4(a)) and for the VBOF (Figure 4(b)) while the vertical lines correspond to the flux values computed by the FBA method. In the first case, FBA returned a zero value for both cases while the distributions were almost identical (Figure 4(a)), implying that the different requirements of host and virus are not related with the TYMSULT reaction. That is the case for the vast majority of the reactions of the model. On the other hand, FBA highlighted that the flux of GK1 increases when VBOF is maximized. That is further highlighted by the flux densities of GK1 reaction where both the mean and the variance of the distribution were increased when VBOF was maximized (Figure 4(b)).

To capture the dependency between the virus' biomass and the flux of the GK1 reaction, a copula of the GK1 and the human biomass fluxes' distributions was computed maximizing first for VBOF. As shown in Figure 5, their negative dependency further support the findings of Renz *et al.*. Interestingly, the FBA flux values for GK1, were rather lower than it is more likely for them to be, both if it is the human biomass that has been maximized or VBOF.

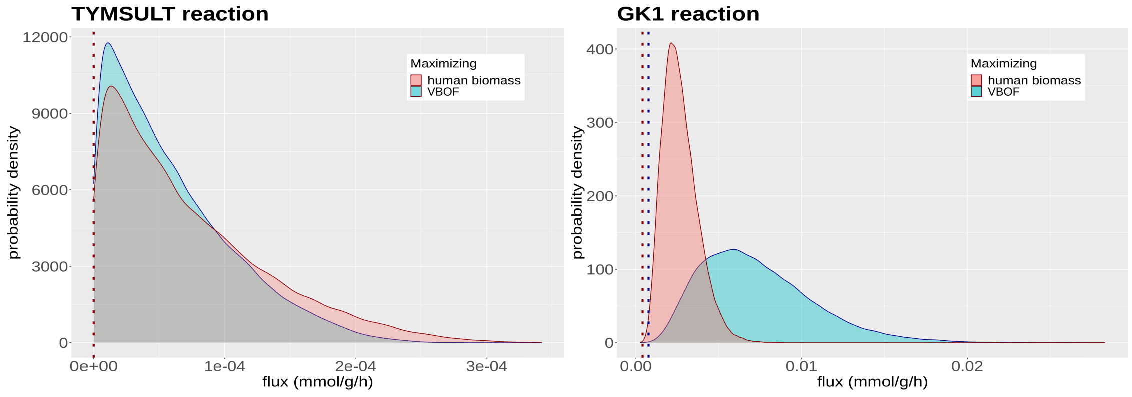

(a) (b)

Figure 4: Distributions of the integrated human-virus model reactions' flux values after maximizing for the host's biomass function (red) and for the VBOF (blue). (a) In case of the TYMSULT reaction, the distribution is the same for both cases (b) Contrary, the flux value distribution of GK1 shifts when maximizing for VBOF.

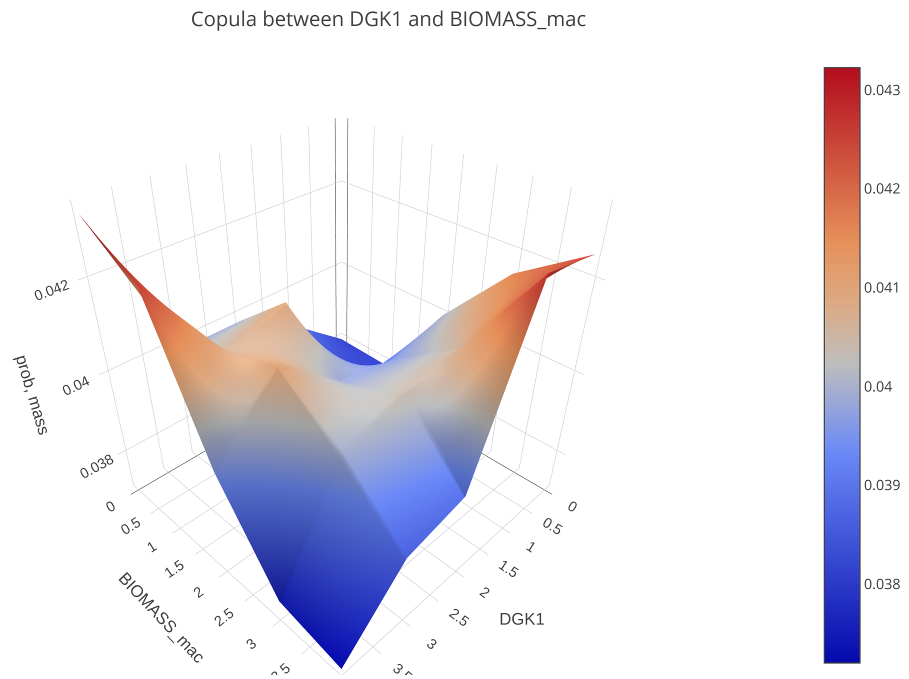

Figure 5: Copula of the biomass function and the GK1 reaction flux values' distributions, after maximizing the integrated model for VBOF. A positive dependency between the two reactions is shown as the flux of the virus biomass is low then most probably, the flux of GK1 is also low and the same applies in case of a high flux value.

In conclusion, flux sampling has the potential to be useful in drug targeting studies [30, 34].

4 Future work

Constraint-based approaches for the analysis of metabolic models may suffer from thermodynamically infeasible loops [33]. As Beard et *al.* first observed [2], metabolic networks can be conceptualized as analogous to current networks, suggesting that Kirchhoff's laws should similarly apply to them. According to Kirchoff, the sum of currents flowing into and out of a junction in a network of conductors is zero (first law, the *current law*) while the total potential difference (voltage) around any closed loop, when considering its direction, must be equal to zero too (second law, the *voltage law*). Mass conservation applied in metabolic modeling ensures the first law. Yet, extra constraints, discussed in [2], need to be applied so the second law is ensured too to avoid flux solutions with active closed loops [33]. Based on previous work [33], `dingo` will support checks for the thermodynamic feasibility of a sample while sampling approaches such as the recently published by Saa et *al.* [32] will be implemented too. Moreover, certain random walks provided by `dingo` —as Billiard walk— can be employed to sample from non-convex bodies [15].

While loopless sampling is strongly related to sampling in non-convex polytopes [32], another common question that rises in so-far implementations is whether it is the uniform distribution the most suitable for investigating a cell's metabolism in an unbiased way. In a previous study, De Martino et *al.* showed that the maximum entropy distribution [10] could be used instead coupled with experimental data for growth. `dingo` will support sampling using the maximum entropy distribution in the near future.

Last, community modelling in microbiome studies is growing. Approaches such as those described in [11] and [3] and implementations such as MICOM [12], COMETS [13] and BacArena [1] have combined community modelling with FBA or dynamic FBA. In a future version of `dingo` it is our intention to support community flux sampling using several community modelling approaches.

References

[1] E. Bauer, J. Zimmermann, F. Baldini, I. Thiele, and C. Kaleta. BacArena: Individual-based metabolic modeling of heterogeneous microbes in complex communities. *PLOS Computational Biology*, 13(5):e1005544, May 2017.

[2] D. A. Beard, S. dan Liang, and H. Qian. Energy balance for analysis of complex metabolic networks. *Biophysical Journal*, 83(1):79–86, 2002.

[3] K. Cerk, P. Ugalde-Salas, C. G. Nedjad, M. Lecomte, C. Muller, D. J. Sherman, F. Hildebrand, S. Labarthe, and C. Frioux. Community-scale models of microbiomes: Articulating metabolic modelling and metagenome sequencing. *Microbial Biotechnology*, 17(1):e14396, 2024. e14396 MICROBIO-2023-392.R1.

[4] A. Chalkis, I. Z. Emiris, and V. Fisikopoulos. A practical algorithm for volume estimation based on billiard trajectories and simulated annealing. *ACM J. Exp. Algorithmics*, 28, may 2023.

[5] A. Chalkis and V. Fisikopoulos. volesti: Volume approximation and sampling for convex polytopes in R, 2020. `https://github.com/GeomScale/volume_approximation`.

[6] A. Chalkis, V. Fisikopoulos, E. Tsigaridas, and H. Zafeiropoulos. Geometric Algorithms for Sampling the Flux Space of Metabolic Networks. In K. Buchin and E. Colin de Verdière, editors, *37th International Symposium on Computational Geometry (SoCG 2021)*, volume 189 of *Leibniz International Proceedings in Informatics (LIPIcs)*, pages 21:1–21:16, Dagstuhl, Germany, 2021. Schloss Dagstuhl – Leibniz-Zentrum für Informatik.

[7] A. Chalkis, V. Fisikopoulos, E. Tsigaridas, and H. Zafeiropoulos. Supplementary files for the dingo python library, Dec. 2023.

[8] Y. Chen, R. Dwivedi, M. J. Wainwright, and B. Yu. Fast MCMC Sampling Algorithms on Polytopes. *Journal of Machine Learning Research*, 19(55):1–86, 2018.

[9] A. Chevallier, S. Pion, and F. Cazals. Hamiltonian Monte Carlo with boundary reflections, and application to polytope volume calculations. Research Report RR-9222, INRIA Sophia Antipolis, France, 2018.

[10] D. De Martino. Scales and multimodal flux distributions in stationary metabolic network models via thermodynamics. *Physical review E*, 95(6):062419, 2017.

[11] C. Diener and S. M. Gibbons. More is different: Metabolic modeling of diverse microbial communities. *mSystems*, 8(2):e01270–22, 2023.

[12] C. Diener, S. M. Gibbons, and O. Resendis-Antonio. Micom: Metagenome-scale modeling to infer metabolic interactions in the gut microbiota. *mSystems*, 5(1):10.1128/msystems.00606–19, 2020.

[13] I. Dukovski, D. Bajić, J. M. Chacón, M. Quintin, J. C. C. Vila, S. Sulheim, A. R. Pacheco, D. B. Bernstein, W. J. Riehl, K. S. Korolev, A. Sanchez, W. R. Harcombe, and D. Segrè. A metabolic modeling platform for the computation of microbial ecosystems in time and space (COMETS). *Nature Protocols*, 16(11):5030–5082, Nov. 2021.

[14] B. García-Jiménez, J. Torres-Bacete, and J. Nogales. Metabolic modelling approaches for describing and engineering microbial communities. *Computational and Structural Biotechnology Journal*, 19:226–246, 2021.

[15] E. Gryazina and B. Polyak. Random sampling: Billiard walk algorithm. *European Journal of Operational Research*, 238(2):497 – 504, 2014.

[16] H. S. Haraldsdóttir, B. Cousins, I. Thiele, R. M. Fleming, and S. Vempala. CHRR: coordinate hit-and-run with rounding for uniform sampling of constraint-based models. *Bioinformatics*, 33(11):1741–1743, 01 2017.

[17] L. Heirendt, S. Arreckx, T. Pfau, S. N. Mendoza, A. Richelle, A. Heinken, H. S. Haraldsdóttir, J. Wachowiak, S. M. Keating, V. Vlasov, et al. Creation and analysis of biochemical constraint-based models using the cobra toolbox v. 3.0. *Nature protocols*, 14(3):639–702, 2019.

[18] J. F. Jadebeck, A. Theorell, S. Leweke, and K. Noh. Hops: high-performance library for non uniform sampling of convex constrained models. *Bioinformatics*, 37:1776–1777, 2021.

[19] F. John. Extremum problems with inequalities as subsidiary conditions. In *Studies and Essays : Courant Anniversary Volume*, pages 187–204. John Wiley & Sons : Interscience Division, New York, 1948.

[20] R. Kannan, L. Lovasz, and M. Simonovits. Random walks and a volume $O(n^5)$ algorithm for convex bodies. *Random Structures and Algorithms*, 11:1 – 50, 1997.

[21] R. Kannan and H. Narayanan. Random walks on polytopes and an affine interior point method for linear programming. *Mathematics of Operations Research*, 37(1):1–20, 2012.

[22] T. C. Keaty and P. A. Jensen. Gapsplit: efficient random sampling for non-convex constraint-based models. *Bioinformatics*, 36(8):2623–2625, 01 2020.

[23] P. Kumar and E. A. Yildirim. Minimum-volume enclosing ellipsoids and core sets. *Journal of Optimization Theory and Applications*, 126(1):1–21, Jul 2005.

[24] L. Lovász, R. Kannan, and M. Simonovits. Random walks and an $O^*(n^5)$ volume algorithm for convex bodies. *Random Structures and Algorithms*, 11:1–50, 1997.

[25] L. Lovász and S. Vempala. Simulated annealing in convex bodies and an $O^*(n^4)$ volume algorithms. *J. Computer & System Sciences*, 72:392–417, 2006.

[26] W. Megchelenbrink, M. Huynen, and E. Marchiori. optgpsampler: An improved tool for uniformly sampling the solution-space of genome-scale metabolic networks. *PLOS ONE*, 9(2):1–8, 02 2014.

[27] W. Megchelenbrink, M. Huynen, and E. Marchiori. optgpsampler: An improved tool for uniformly sampling the solution-space of genome-scale metabolic networks. *PLOS ONE*, 9(2):1–8, 02 2014.

[28] V. D. Milman and A. Pajor. Isotropic position and inertia ellipsoids and zonoids of the unit ball of a normed n-dimensional space. *Geometric aspects of functional analysis*, pages 64–104, 1989.

[29] B. Niebel, S. Leupold, and M. Heinemann. An upper limit on gibbs energy dissipation governs cellular metabolism. *Nature Metabolism*, 1(1):125–132, 2019.

[30] N. D. Price, J. Schellenberger, and B. O. Palsson. Uniform sampling of steady-state flux spaces: means to design experiments and to interpret enzymopathies. *Biophysical journal*, 87(4):2172–2186, 2004.

[31] A. Renz, L. Widerspick, and A. Dräger. FBA reveals guanylate kinase as a potential target for antiviral therapies against SARS-CoV-2. *Bioinformatics*, 36(Supplement_2):i813–i821, Dec. 2020.

[32] Saa. LooplessFluxSampler: An efficient toolbox for sampling the loopless flux solution space of metabolic models. 2023.

[33] P. A. Saa and L. K. Nielsen. ll-ACHRB: a scalable algorithm for sampling the feasible solution space of metabolic networks. *Bioinformatics*, 32(15):2330–2337, 03 2016.

[34] J. Schellenberger and B. Ø.. Palsson. Use of randomized sampling for analysis of metabolic networks. *Journal of biological chemistry*, 284(9):5457–5461, 2009.

[35] R. L. Smith. Efficient Monte Carlo procedures for generating points uniformly distributed over bounded regions. *Operations Research*, 32(6):1296–1308, 1984.

[36] M. J. Todd and E. A. Yıldırım. On khachiyan's algorithm for the computation of minimum-volume enclosing ellipsoids. *Discrete Applied Mathematics*, 155(13):1731–1744, 2007.

[37] K. Van den Meersche, K. Soetaert, and D. Van Oevelen. xsample(): An r function for sampling linear inverse problems. *Journal of Statistical Software, Code Snippets*, 30(1):1–15, 2009.
